# Better primer design for metagenomics applications by increasing taxonomic distinguishability

**DOI:** 10.1186/1753-6561-7-S7-S4

**Published:** 2013-12-20

**Authors:** Melita Jaric, Jonathan Segal, Eugenia Silva-Herzog, Lisa Schneper, Kalai Mathee, Giri Narasimhan

**Affiliations:** 1 Bioinformatics Research Group (BioRG), School of Computing and Information Sciences, Florida Intl. Univ., Miami, FL 33140, USA; 2 Department of Molecular Microbiology and Infectious Diseases, Florida International University, Miami, FL 33140, USA

## Abstract

Current methods of understanding microbiome composition and structure rely on accurately estimating the number of distinct species and their relative abundance. Most of these methods require an efficient PCR whose forward and reverse primers bind well to the same, large number of identifiable species, and produce amplicons that are unique. It is therefore not surprising that currently used universal primers designed many years ago are not as efficient and fail to bind to recently cataloged species. We propose an automated general method of designing PCR primer pairs that abide by primer design rules and uses current sequence database as input. Since the method is automated, primers can be designed for targeted microbial species or updated as species are added or deleted from the database. *In silico *experiments and laboratory experiments confirm the efficacy of the newly designed primers for metagenomics applications.

## Introduction

DNA extraction and PCR amplification are essential steps in a number of different applications including forensics, sequencing, metagenomic analyses (i.e., study of community profiles) and comparison of ecosystems using their microbial profiles [1]. Design of primer pairs used in PCR depends on the target application and can be specific to a particular species, gene, ortholog group, taxon or community. In projects that aim to discover community composition and structure, primers are mainly required to (1) be *universal *in their ability to bind to a maximum number of different *target *species and (2) produce maximally **distinguishable **amplicons for the taxa of interest, which is the basis for the post PCR data analyses and species identification.

Launched in 2008, the Human Microbiome Project (HMP) [2] is the project *du jour *for studying microbial community composition and structure in different environmental niches within the human body. HMP primarily aims to understand the differences in the microbial composition between unhealthy and healthy states of five body sites: oral, skin, vaginal, gut and nasal/lung. HMP studies often utilize universal primers that amplify regions of the 16S rRNA, a popular target gene for studying taxonomic and evolutionary relationship between microbial organisms [3,4]. However, universal primers were developed over 20 years ago [5-7]. Since then many new bacterial and archaeal species have been discovered [8], so much so that a number of 16S rRNA databases have been created and updated many times. (RDP [9] has been updated 31 times in the last 5 years and has roughly quadrupled in size; Silva [10] with 16 full releases in 6 years, has grown six-fold with 449 new species in the last release.) Inevitably the universal primers have become too specific/biased, i.e., they fail to extract newly added species from the current database. Necessitated by particular projects, a number of taxon-specific primer pairs [11,12] have been developed and efforts have been made to improve complementarity of the majority of universal primers [13-15].

In this paper, primer design is cast as a constrained optimization problem, in which the pairs of primers must satisfy a range of criteria for being functional, while maximizing the distinguishability with respect to a given set of microbial organisms. Experimental results show the efficacy of the proposed method.

## Background and motivation

In PCR, forward and reverse primers hybridize to specific locations on the target DNA sequence and the fragment between the primer binding sites is amplified. Efficient and effective PCR requires that the primers satisfy a set of widely accepted interdependent conditions (For example, see http://www.premierbiosoft.com/tech_notes/PCR_Primer_Design.html): (1) melting temperatu-re (*T_m_*) range, (2) primer specificity, (3) GC clamp, (4) primer-primer interactions, (5) bounded degeneracy, and (6) amplicon length. When primers hybridize to multiple locations, the efficiency of PCR is reduced. PCR efficiency depends greatly on the strength of hydrogen bonds formed between the primer and the template. The distinction between a strong base (C or G) and a weak base (A or T) is based on the number of hydrogen bonds (3 and 2, respectively) that a base requires to bind to the reverse strand. Primers with greater GC content result in stronger anchoring and greater energy is needed to break the bonds. Given that the PCR elongation happens at the 3' end, it is important that 3' end anchors well and better (more strongly) than the 5' end. Still, it is recommended that the 3' end have at most two strong bases. PCR works efficiently only within the temperature range defined by the primers it uses. Thus, the temperature ranges defined by the forward and reverse primers must overlap. At higher temperatures primer-template binding is more specific and it is harder for primers to form secondary structures. However, the primer-template bonds that are formed are broken more easily, which reduces PCR efficiency. Hence the need for high GC content. At low temperatures, although the primer-template bonds are not easily broken, primers hybridize more easily. Furthermore, efficiency of PCR is greatly diminished by allowing forward or reverse primer to either bind to itself (selfDimer) or to each other (primerDimer). It is particularly important that the 3' ends of forward and reverse primers do not create primer dimers. This is implicitly taken care of by the rule of not having more than two strong bases at the 3' end. High primer degeneracy could also be another factor contributing to decreased primer efficiency. A degenerate primer is a mixture of non-degenerate primers. Hence, the increase in primer degeneracy is proportional to decrease in concentration of the individual primers.

Our studies show that most of the universal primers and their derivatives in current use do not abide by *all *of these primer design rules. In Table 1, the primers whose IDs start with U are the universal primers. Violations of the rules governing optimal primer design are underlined and in bold font. The column labeled *nHits *indicates the number of RDP database sequences that the primer is likely to anneal to. For PCR to produce enough amplicon for sequencing or for metagenomic analysis this number must be as high as possible. The two best universal primers in the list, U518R and U337F, do not have a compatible reverse primer. The first one is always paired with U8F to amplify the V13 region (e.g., HMPV13 primer pair). However, U8F has a very low number of hits from RDP. That is because the sequenced data in the RDP database often does not provide sequence data in that region. The U337F primer does not have compatible reverse primer, since its temperature range does not overlap with any of the universal reverse primers (U805R, U907R, U1492R).

**Table 1:** Universal Primers and their Variants [16,17]

primerID	5'→3' seq	deg	sDimer	*T_m _*range	**GC****% range**	length	nHits
U8F	AGAGTTTGATCCTGGCTCAG	1	4	51.78→51.78	0.50→0.50	20	** 1553 **

U336R	ACTGCTG**CSYCCC**GTAGGAGTCT	4	4	60.62→62.40	0.61→0.65	22	** 7408 **

U337F	GACTCCTACGGGAGGCWGCAG	2	6	60.21→60.21	0.67→0.67	21	7689

U518R	GTATTACCGCGGCTGCTGG	1	6	55.41→55.41	0.63→0.63	18	8417

U533F	GTGCCAGCMGCCGCGG**TAA**	2	6	57.56→59.72	0.68→0.74	19	8929

U785F	**GG**ATTAGATACCCTGG**TA**	1	4	** 45.77→45.77 **	** 0.44→0.44 **	18	8183

U805R	GACTACCAGGGTATC**TAAT**C	1	4	49.73→49.73	** 0.45→0.45 **	20	8171

U907R	**CCG**TCAATTCCTTTRAG**TTT**	2	4	** 45.63→47.68 **	** 0.35→0.40 **	20	** 7296 **

U928F	TAAAACTYAAAKGAATTGACGGG	4	4	48.14→51.71	** 0.30→0.39 **	23	** 5090 **

U1100F	YAACGAGCGCAACCC	2	4	** 44.67→47.41 **	0.60→0.67	** 15 **	8980

U1492R	**GG**TTACCTTGTTACGAC**TT**	1	3	46.77→46.77	** 0.42→0.42 **	19	** 449 **

HMPV1F	GAGTTTGATCCTGGCTCAG	1	4	51.09→51.09	0.53→0.53	20	** 1778 **

HMPV3R	ATTACCGCGGCTGCTGG	1	6	51.88→51.88	0.65→0.65	17	8428

HMPV3F	**CC**TACGGGAGGCAGCAG	1	3	54.29→54.29	0.71→0.71	17	8735

HMV5R	**CCCG**TCAATTCMTTTRAGT	4	4	44.62→48.93	** 0.37→0.47 **	19	8533

HMPV6F	**G**YAACGAGCGCAACCC	2	4	48.50→51.06	0.63→0.69	** 16 **	8507

HMPV9R	AAGGAGGTGATCCAGCCGCA	1	4	55.88→55.88	0.60→0.60	20	** 1193 **

N337F	**GG**AGGCAGCAGTRRGG**AAT**	4	2	51.09→55.41	0.53→0.63	19	8570

N775R	CTACCRGGGTATCTAATCC	2	4	48.93→51.09	0.47→0.53	19	8427

K331F	**CC**TACGGGNGGCWGCAG	8	6	54.29→56.70	0.71→0.76	17	8882

K775R	GACTACHVGGGTATCTAATCC	9	** 10 **	50.45→54.36	** 0.43→0.52 **	21	8868

S899R	**CCG**TCAATTYMTTTRAGT	8	4	** 38.93→45.77 **	** 0.28→0.44 **	18	8779

HMP commissioned a study that produced a set of recommended primer pairs, their experimental settings and post experimental data processing and analysis workflow [17]. The resulting primers, shown in Table 1, whose ID starts with HMP, are variants of universal primers, with degeneracy increased so as to increase the number of hits in the database, which in turn allows for more precise sequenced read classification and hence better estimation of sample diversity and richness, both of which are fundamental measures used in metagenomic studies.

The recent ubiquity and affordability of HMP-supported studies is due to the advent of Next Generation Sequencing (NGS) technologies that produce large volumes of sequenced reads cheaply. (Note that even though NGS makes it possible to cheaply sequence the entire genome instead of just the 16S region, it may not much help distinguishability of the microbes in the community because of the proliferation of highly conserved genes and the presence of horizontally transferred genes between members of the community.) NGS costs are affordable, but they are not low enough to allow for an experiment to be repeated a number of times. Thus, *in silico *studies have been conducted to assess the diversity of the amplicons using universal and HMP primers [4,18-20]. Although primers such as UV34, HMPV35 and UV6 are predicted to result in high diversity of the amplicons, they are flawed and can be improved.

We see two ways of improving metagenomic studies. First, we propose an automated, general method of designing new primers based on the current content of a target database (Table 2) that abide by primer design rules (Table 3). Since the method is automated, it will be easy to update the primers as new species are added to the database or a different target set is selected from the database. Second, we observe that different species are distinguishable by different variable regions. Given, the affordability of NGS sequencing, and the difficulty in designing primers that are both, universal and abide by primer design rules, we propose the use of multiple sets of primer pairs. These sets are automatically chosen so as to maximize the number of distinguishable species. In this paper we present a software package that achieves both objectives.

## Algorithm

We start with some required notation. Let S  denote a set (database) of sequences over the alphabet Σ = {A, C, G, T, R, Y, S, W, K, M, B, D, H, V, N}, which corresponds to the letters used in the IUPAC code (http://www.bioinformatics.org/sms/iupac.html). Each letter in Σ corresponds to a set of bases from the DNA alphabet {A, C, G, T}. For example, the letter H corresponds to the set {A, C, T}. Each letter of Σ  has a *degeneracy *value, which equals the cardinality of its base set. We denote this by DEG(*a*), for a∈Σ. The *degeneracy *of a sequence *S *is defined as the product of the degeneracy values of each of its letters. More formally, $\text{DEG}(S)\;=\;\overset{l}{\underset{k=1}{\prod }}\text{DEG}(S\left(k,1\right))$, where *S*(*j*, *l*) is a contiguous subsequence of length *l *starting at position *j *in *S*. For example, let *S*_1 _= AAGGATCG, *S*_2 _= WGSANS, and *S*_3 _= AGGSTD, then DEG(*S*_1_) = 1, DEG(*S*_2_) = 32 and DEG(*S*_3_) = 6. A sequence is called *non-degenerate *if its degeneracy equals 1. Degeneracy of a sequence is therefore a measure of the number of different non-degenerate sequences that it represents (or matches).

**Match**. A letter, *p*, from a primer sequence is said to MATCH a letter, *t*, from a target sequence, if the set of bases corresponding to *t *is a subset of the set of the bases corresponding to *p*. Thus, for example, if the target base is R and the primer base is D then MATCH(D, R) = *true*, while match(R, D) = *false*. On the other hand, MATCH(G, H) = MATCH(H, G) = *false*. Extending this definition to sequences, a primer sequence *P *of length *j *is said to match a target sequence *T*, if there exists a location *l*, such that the letters of *P *match the corresponding letters of *T *(*j, l*). For example, for the sequences mentioned above, MATCH(*S*_2_, *S*_3_) = *false*, while MATCH(*S*_2_, *S*_1_) = *true *since *S*_2 _*matches *the subsequence of *S*_1 _of length 6 starting from location 2. Biologically, if a primer sequence *matches *a target sequence, then the primer sequence would hybridize to the target sequence at the location of the match.

Degenerate codes such as S = {C, G}, W = {A, T} and N = {A, C, G, T} mean different things in different contexts. A degenerate code N in the database (e.g. RDP) means that the value of that base is not precisely determined. Thus, an N suggests that the base is A or C or G or T and any choice of the base (other than N) for the primer at that position could fail to match. On the other hand, a degenerate code in the primer means that all possible bases are present in the primer at that position. Thus, an N suggests that the base is A and C and G and T, i.e., different copies of the primer with different bases in that position are used. Consequently, it would match any letter in the corresponding target location.

**Common**. Two letters from Σ  are said to be in COMMON if the sets of bases they represent have a non-empty intersection. Thus, for example, COMMON(R, D) = TRUE, while COMMON(G, H) = *false*. The definition can be extended to two sequences being in common as follows: Two sequences are said to be in COMMON if every letter of one of the sequences is in common with the corresponding letter from the other sequence.

**Design parameters for optimal degenerate primers**. As mentioned earlier, the PCR reaction requires a number of conditions to be satisfied by the primers in order to work well. The following parameters and their constraints have been compiled from various design manuals. (For example, see http://www.premierbiosoft.com/tech_notes/PCR_Primer_Design.html.) We will refer to them henceforth as the **Optimal Primer Design Rules**.

**(1) **SELFDIMER(*P*) is defined as the length of the longest contiguous COMMON subsequence between the primer *P *and its reverse complement. Good primer design requires that this quantity be minimized.

**(2) **PRIMERDIMER(*F, R*) is defined as the length of the longest contiguous COMMON subsequence between the forward (*F *) and the reverse (*R*) primers. This quantity should be minimized.

**(3) **AMPLICONLENGTH(*F, R*) is the length of sequence that starts with *F *and finishes with *R*. The amplicon length is defined in the range of 150-600 bp. The lower limit prevents amplification of conserved regions. The upper limit is determined by the current read length that sequencing technologies support.

**(4) **RUNLENGTH(*P *) is the maximum number of consecutive occurrences of the same base from the set {A, C, G, T}. Thus, for the U336R primer in Table 1, the red colored subsequence has RUNLENGTH(U336R) = 6.

**(5) **CG%(*P *) is the percent of {C, G} bases in P. If DEG(*P *) = 1 or if only S or W degenerate bases are present then there is no ambiguity in the computation of CG%. Since other degenerate bases cause ambiguity in the count, we end up with a range of values [MINCG%(*P*), MAXCG%(*P*)]. Good primer design requires that CG%(*P*) be between 50 and 65. In practice, this constraint may be slightly relaxed.

**(6) **While many methods exist in the literature, TM(*P *) is calculated by the *Basic T_m_ equation *(see http://www.promega.com/techserv/tools/biomath/calc11.htm): T*m*(*P*) = 64.9 + 41 * (C*G*%(*P*) - 16.4)*/length*(*P*). If DEG(*P*) = 1, or if only S or W degenerate bases are present then there is no ambiguity in the above calculation. As with CG%, if other degenerate bases are present in the primer sequence, then a range of values is possible, and is denoted by [MINTM(*P*), MAXTM(*P*)]. Good primer design requires that the entire range lies between 55° and 65°. It also requires that RANGETM(*P*) = MAXTM(*P*)-MINTM(*P*) not be more than 5°. Again, in practice, a slight deviation from these conditions is acceptable.

Instead of allowing an arbitrary sequence to be a candidate primer, we define some more terms that help us narrow down the search space for our candidate primers.

**Viable Primer**. A *viable primer *is a sequence that satisfies the optimal primer design rules from above.

**Common Viable Primer for ℋ **. A common viable primer for a multiset of sequences ℋ  is a viable primer if it *matches *at least *minSupport *number of sequences in ℋ .

**Derivative**. A sequence *P *is derived from a sequence *T *if MATCH(*P*, *T*) = *true*. (For example, *P*_1 _= AWGCT is a derivative of *T *= ATGCT, but *P*_2 _= ATGGT is not.)

**Amplicon from sequence ***S ***with Primer Pair **(*F*, *R*). An amplicon *A *from sequence *S *is a contiguous subsequence of *S *defined by a *viable *primer pair (*F*, *R*). *A *must start with a subsequence that matches the reverse complement of the forward primer *F *and must end with a subsequence that matches the reverse primer *R*.

**Reference Target Sequence ***T*. A sequence *T *from a database of sequences S  is said to be a reference target sequence if the forward and reference primers are derived from *T *.

### Problem statement

The primary task of a viable primer is to *match *as many sequences as possible from a target database. For primer pairs, it is important to maximize the number of sequences that match *both *primers. In applications such as metagenomics, where the amplicons are sequenced and then used to identify the organism from which it originated, a third requirement is that amplicons generated by the primers be uniquely distinguishable.

**The Degenerate Primer Design Problem**. For a given set of parameters P , a set of sequences S  and a reference target sequence T∈S, find a viable primer pair (*F*, *R*) that matches *T *for which the number of distinguishable amplicons generated by its primers (*F*, *R*) for the sequences in S  is maximized.

In other words, given a database S  (e.g., RDP), a reference sequence *T *(e.g, *E. coli*), and values for the design parameters, P , including MAXSELFDIMER, MAXPRIMERDIMER, MAXRUNLENGTH, MINCG%, MAXCG%, MINTM, and MAXTM, develop a set of viable forward and reverse degenerate primer pairs with the maximum number of unique amplicons.

### Algorithm description

$$\begin{array}{l} \begin{array}{cc} \bar{Algorithm\;1\;\text{S}\text{ELECT}\text{ P}\text{RIMER}\text{ C}\text{ANDIDATES}\;} & \end{array}\\ \bar{\;Input:\;S:\;\text{sequence database; }T:\;\text{sequence;}\;P:}\\ \;\text{primer design parameters}\\ \;Output:ℱ/ℛ:\text{primer list of all possible degen-}\\ \;\text{erate forward or reverse primers}\\ \;\\ \;ℱ=\emptyset \\ \;for\;pos=1:\text{length}(T)\;do\\ \;\;for\;len=P.\text{MIN}\text{L}\text{ENGTH}:P.\text{MAX}\text{L}\text{ENGTH}\;do\\ \;\;\;P=T(pos,len)\\ \;\;\;if\;\text{IS}\text{V}\text{IABLE}(P,P)\;then\\ \;\;\;\;ℋ=\emptyset ;\\ \;\;\;\;ℋ\leftarrow \text{FIND}\text{B}\text{EST}\text{V}\text{ALID}\text{M}\text{ATCH}(S,P,P)\\ \;\;\;\;ℱ\leftarrow \text{DEVELOP}\text{D}\text{EG}\text{P}\text{RIMER}(ℋ,P)\\ \;\;\;end\;if\\ \;\;end\;for\\ \underline{\;end\;for\;} \end{array}$$

The reference sequence *T *ensures that the number of possible primer sequences is finite. The observation below prunes the search space further.

**Observation 1 ***If primer P*_1 _*is not viable then a primer P*_2 _*derived from P*_1 _*is not viable either*.

Also, given primers *P*_1_, *P*_2_, such that DEG(*P*1) *≤*DEG (*P*_2_) and MATCH(*P*_1_, *P*_2_) = *true*, then (a) SELFDIMER(*P*_1_) *≤ *SELFDIMER(*P*_2_), (b) TEMPRANGE(*P*_1_) *≤ *TEMPRANGE(*P*_2_), (c) CG%(*P*_1_) *≤ *CG%(*P*_2_), and (d) RUNLENGTH(*P*_1_) *≤ *RUNLENGTH(*P*_2_).

**Scanning for Candidate Forward and Reverse Primers**. To make the search more efficient, we focus on primer sequences derived from the *target *sequence. Observation 1 is also used to prune away candidate primers that are not viable.

**Algorithm **SELECTPRIMERCANDIDATES shown here designs set ℱ  of viable common degenerate forward primers that correspond to all viable primers *P *from reference sequence *T *and sequence database S . A similar design is performed to generate the set of viable common reverse primers ℛ . First, for each viable primer from *T *function FIND-BESTVALIDMATCH generates a set ℋ  of *unique best valid matches *in S . Note that not all target sequences contribute to ℋ . Some target sequences might not have a *valid match *or *unique best valid match *for the relevant subsequence of *T*.

$$\begin{array}{l} \begin{array}{cc} \bar{Algorithm\;2\;\text{DEVELOP}\text{D}\text{EG}\text{P}\text{RIMER}\;} & \end{array}\\ \bar{\;Input:ℋ:\text{set of hits; }P:\text{primer design parameters}}\\ \;Output:D:\text{list of degenerate primers}\\ \;\\ \;D=\emptyset ;\text{cnt}=0\\ \;\text{profileMtx}=\text{CREATE}\text{P}\text{ROFILE}\text{M}\text{TX}(ℋ)\\ \;if\;\text{INIT}\text{C}\text{HECK}(\text{profileMtx},P)\;then\\ \;\;\text{currentPrimer}=\text{INIT}\text{P}\text{RIMER}(\text{profileMtx})\\ \;\;while\;\text{IS}\text{V}\text{IABLE}(\text{currentPrimer},P)do\\ \;\;\;\text{degPrimer}=\text{currentPrimer}\\ \;\;\;\text{cnt }+=ℋ.\text{REMOVE}\text{D}\text{EG}\text{M}\text{ATCH}(\text{degPrimer})\\ \;\;\;if\;\text{cnt}\geq \min\text{Support }then\\ \;\;\;\;\text{Add degPrimer to }D\\ \;\;\;end\;if\\ \;\;\;\text{profileMtx}=\text{CREATE}\text{P}\text{ROFILE}\text{M}\text{TX}(ℋ)\\ \;\;\;\text{PQ}\leftarrow \text{entries of profileMtx prioritized by the entry}\\ \;\;\;\text{currentPrimer}=\text{degPrimer}\cup \text{PQ.pop}()\\ \;\;\;while\;!\text{IS}\text{V}\text{IABLE}(\text{currentPrimer},P)\wedge !\text{PQ.isEmpty}()do\\ \;\;\;\;\text{currentPrimer}=\text{degPrimer}\cup \text{PQ.pop}()\\ \;\;\;end\;while\\ \;\;end\;while\\ \underline{\;end\;if\;} \end{array}$$

**Unique best valid match between a primer and a sequence**. A *match *between a viable primer *P *and a sequence *S_i _*is *valid *if there exists a contiguous subsequence in *S_i _*of the same length as *P *and that has at most *log*_2_(MAXDEGENERACY) mismatches with *P*. If there is only one *valid match *between *P *and *S_i _*that has the least number of mismatches then that is the *unique best valid match*.

**Algorithm **DEVELOPDEGPRIMER develops a set of *viable common primers *corresponding to input multiset ℋ . From the aligned set of hits ℋ , we create a frequency count matrix for each position. If the highest frequency in a position (column) in the alignment is smaller than *minSupport*, then that position in the primer would require to be degenerate. We first check that the number of positions that require degeneracy is no greater than *log*_2_(MAXDEGENERACY). If it is, then the bound on degeneracy would be violated and we exit without computing a *common *degenerate primer. Otherwise, we construct the initial degenerate primer, *degPrimer*, with each of its bases set to the base that occurs most frequently in that position. If the initial degenerate primer is not a viable primer we exit since its derivates need not be considered by Observation 1.

$$\begin{array}{l} \begin{array}{cc} \bar{Algorithm\;3\;\text{D}\text{ESIGN}\text{P}\text{RIMER}\text{P}\text{AIR}\;} & \end{array}\\ \bar{\;Input:S:\text{sequence database};\;ℱ,ℛ\;\text{forward},\;\text{reverse primer sets}}\\ \;\text{from SelectPrimerCandidates};P\;\text{primer pair design parameters}\\ \;Output:A\;\text{list of primer pairs};AP\;\text{list of pair of primer pairs}\\ \;\\ \;A=\emptyset \\ \;ℱ=\text{SORT}\text{P}\text{ER}\text{R}\text{GN}(ℱ),ℛ=\text{SORT}\text{P}\text{ER}\text{R}\text{GN}(ℛ)\\ \;for\;i=1:numRgns(ℱ),j=1:numRgns(ℛ)\;do\\ \;\;A\leftarrow \text{MATCH}\text{B}\text{EST}\text{P}\text{ER}\text{R}\text{GN}\text{FR}(ℱ(i),\;ℛ(j),P)\\ \;end\;for\\ \underline{\;A,AP\leftarrow \text{FIND}\text{D}\text{ISTINGUISH}\text{T}\text{OTAL}(S,A)\;} \end{array}$$

Starting from the initial primer, we remove all entries of ℋ  that match the current primer. To increase the number of hits in ℋ , we use a greedy method to iteratively increase primer degeneracy, one base at a time, as follows. In each iteration, from the remaining sequences in ℋ , we reconstruct the frequency count matrix, storing all positions that do not yet match the current primer, ordered by the frequency count of the highest frequency. We choose to increase degeneracy of a base with the highest frequency count, while making sure that the new primer remains viable. Ties are broken in favor of the base for which the increased degeneracy causes the least increase in overall degeneracy of the sequence. When a new common viable primer is found, it is added to the candidate primer set ℱ .

**Algorithm **DESIGNPRIMERPAIR takes a set of viable forward primers and a set of viable reverse primers, groups each set into subregions, sorting each group by their number of hits in the database. To allow for better primer pairing with regard to distinguishabilty, each conserved region is further subdivided into subregions, defined by the primer start location. The sorted lists of forward and reverse primers are scanned to select the pair with maximum number of simultaneous hits in the database that abide by optimal design rules. Once we have primer pairs and their number of hits in the database, we focus on amplicon distinguishability. In the context of degenerate primer design, an amplicon is distinguishable if and only if it has a *match *to exactly one sequence in the database. In order to select sets of candidate primer pairs, we extend the notion of amplicon distinguishability to two or more primer pairs. A sequence is distinguishable if it produces a distinguishable amplicon with at least one of the primer pairs in the chosen set.

**Function **FINDDISTINGUISHTOTAL creates a taxon matrix to easily gather and compare results for each primer pair and across two (or more) primer pairs. Data can be generated for any combination of two primer pairs. It is possible to request only a number of best combination for a desired metric (distinguishable/total number of hits per genus/species).

## Results

*In silico *design of degenerate primers

We run the algorithm on sequences from the 16S rRNA RDP database (downloaded on February 12, 2012). The set is described in Table 2. We used the *E. coli *sequence as the template sequence and the set of parameters used for primer design and primer pair design are described in Table 3. The algorithm described above found 208 (244, resp.) *viable common *forward (reverse, resp.) primers from 8 forward and (10 reverse, resp.) regions. We note that there is no *viable common *primer in the conserved regions between variable regions one and two. Note that our procedure was able to identify the widely used universal primers, U337F and U518R, which abide by the optimal design rules. The second primer in Table 4 in region FV3 is a more degenerate version of U337F, and the second primer under the region RV3 is a degenerate version of U518R.

**Table 2:** RDP Sequence set

Number of Sequences	9175
Number of Species	8372

Number of Genera	1779

Number of Phyla	29

Avg Sequence Length	1468

Max Sequence Length	1847

Min Sequence Length	1225

Strain	Typed

Size	≥ 1200

Source	Isolates

Quality	Good

**Table 3:** Primer Design Rule Parameters

Design Parameters	Values Used
allowable 3' ends	S[SW,WS][SW,WS,SS]

allowable 5' ends	WW, [WS,SW]SW

MINTEMP	48

MAXTEMP	70

MAXTEMPRANGE	8

MINCG%	40

MAXCG%	60

MINPRIMERLENGTH	17

MAXPRIMERLENGTH	24

MAXDEGENERACY	64

MAXSELFDIMER	6

MAXPRIMERDIMER	6

MAXRUNLENGTH (bp)	4

MINAMPLICONLENGTH	150

MAXAMPLICONLENGTH	600

**Table 4:** Select Designed Primers

regionID	5'→3' sequence	deg	sDimer	TmRange	**CG**%**Range**	nHits	pos*Ecoli*
FV3	ACWCCTRCGGGWGGCWG	16	4	51.88→54.29	0.65→0.70	8867	345

FV3	ACWCCTRCGGGWGGCWGCAG	16	6	57.93→59.98	0.65→0.70	8854	345

FV4	AGCAGCCGCGGTAANACG	4	6	52.60→54.88	0.61→0.67	8305	528

FV6	WACSCGMRGAACCTTACC	16	6	48.04→52.60	0.50→0.61	8045	975

FV7	ATGGYYGTCGTCARCTCG	8	4	48.04→54.88	0.50→0.67	8821	1062

FV7	AGTCCNRYAACGAGCGCAACC	16	4	54.36→60.21	0.52→0.67	8788	1103

FV8	AGGAAGGHGDGGAYGASGTC	36	6	53.83→59.98	0.55→0.70	8810	1185


RV3	TTACCGCGGCTGCTGGCAC	1	6	57.56→57.56	0.68→0.68	8926	505

RV3	TWTYACCGCGGCTGCTGG	4	6	52.60→54.88	0.61→0.67	8543	508

RV4	ACCAGGGTATCTAAKCCTG	2	4	48.93→51.09	0.47→0.53	8292	773

RV6	TYACRRCACGAGCTGWCG	16	5	48.04→54.88	0.50→0.67	9026	1052

RV7	ACGTCRTCCHCWCCTTCC	12	4	50.32→54.88	0.56→0.67	8114	1166

RV8	TGWGTACAAGRYCCGRGAACG	16	6	52.40→58.26	0.48→0.62	8411	1367

Note that our algorithm found a forward primer better than U337F in the FV3 region with more hits and and lower SELFDIMER value. Similarly, a better reverse primer was found in the RV3 region with almost 400 more *matches *in RDP. Table 4 represents only a sample of the *viable common primers *found by the algorithm and represent the best primers in terms of the number of RDP hits. Table 4 shows a number of FV7 primers to illustrate that within a region two primers can have significantly different parameters, but still are valid options in the PRIMERPAIRDESIGN. In Table 5 we include only a selection of designed primer pairs, together with select primer pairs from HMP and Universal primer pairs for comparison. Comparing primer pairs spanning roughly the same regions, we note that primer pair F528-R1052 is better than HMP_V3-5 by most measures. Pair F345-R505 improves upon U337F-518R, and pair F345-R775/873 compares favorably to U337F-805R. Pairs F1064-R1367 or F1185-R1367 are by most measures better than HMPV69. Primer F1064 is a better substitute for U1100F primer, used as HMPV6F. The table also shows that different primer pairs can be chosen depending on what features are important to a user. For example, for region V8, primer pair F1185-R1367 would be chosen if phylum distinguishability is considered important, but not if genus or species distinguishability is considered important. Furthermore, the results also indicate that total number of hits per taxon and the corresponding distinguishability do not necessarily correlate (compare F528-R1052 and F797-R1052). The same can be said for the distinguishability between genus and species (compare F345-R922 and F528-R775).

**Table 5:** Comparison of Primer Pairs

Primer Pair Parameters			Total Number of Hits per Taxon				Number of Distinguishable Amplicons per Taxon		
**PrimerPairID**	**TmRange**	**Dimer**	**Strain**	**Species**	**Genus**	**Phylum**	**Species**	**Genus**	**Phylum**

F345-R1052	6.83	4	8738	7961	1702	29	7110	1696	29

F345-R505	5.69	4	8654	7887	1684	27	4726	1554	27

F345-R775	5.18	4	7981	7342	1565	24	5943	1549	24

F345-R873	6.47	5	7961	7264	1537	27	6031	1527	27

F345-R922	6.83	5	7740	7074	1452	22	5893	1443	22

F528-R1052	6.83	4	8212	7520	1647	27	6295	1639	27

F797-R1052	6.83	3	8399	7720	1639	25	5396	1610	25

F1064-R1367	6.48	4	8155	7439	1596	27	4926	1534	27

F1185-R1367	7.58	4	8099	7375	1568	28	3776	1407	28


HMPV35	9.67	5	8174	7452	1573	24	6225	1564	24

HMPV13	0.79	3	1572	1198	498	18	1104	497	18

HMPV69	7.38	3	1162	818	304	20	646	301	20

U337F-805R	10.48	3	7011	6479	1405	17	5218	1392	17

U337F-518R	4.81	6	7059	6512	1429	20	3886	1325	20

NossaV34	6.47	4	8042	7413	1546	17	5997	1533	17

To see whether and how amplicon length is correlated taxon distinguishability we ran the *degenerate primer design *algorithm with no upper bound for amplicon length. One may hypothesize that an increase in the number of variable regions in the amplicons generated by a primer pair will lead to an increase in taxon distinguishability. The best result was obtained with the first primer pair in Table 5 (F345-R1052). It flanks V3-V6 regions. Hence increase in amplicon length does not necessarily increase taxon distinguishability. We postulate that conserved regions of different species are not uniformly conserved. This brings us to the final step: combination of different primer pairs that extract different taxons to increase taxon distinguishability. We include HMP and universal primers from Table 5 in the input data set. The results are presented in Table 6 below. In Table 6 the first two pairs of primer pairs are the best combination for distinguishing the most number of species and most number of genera, respectively, if amplicon length is not bounded.

**Table 6:** Pairs of Primer Pairs with number of distinguishable species, genera and phyla.

PrimerPair1	PrimerPair2	Species: Distinguish/Total	Genus: Distinguish/Total	Phylum
V36-F348-R1050	V48-F532-R1367	**7635**/8284	1745/1763	29

V36-F348-R1042	V47-F528-R1164	7537/8256	**1757**/1758	29


HMPV35	V46-F528-R1052	7270/8217	1737/1742	28

V34-F345-R775	V46-F528-R1052	7210/8091	1727/1734	29

V3-F348-R496	V46-F528-R1052	7016/8205	1746/1754	28

HMPV35	V58-F797-R1367	7340/8083	1689/1692	28


V3-F345-R505	V78-F1064-R1367	6760/8228	1734/1743	29

V3-F345-R505	V57-F797-R1166	6266/8227	1712/1745	29

V3-F348-R496	V46-F528-R1052	7016/8205	1746/1754	28

If we allow amplicons of arbitrary lengths then designed primers can generate and distinguish all the Genera and can generate all the Species, while failing to distinguish 563. As the lengths of the sequenced reads are increased, the number of taxons not generated or distinguished by two primer pairs gets smaller. In particular, note that if we do not bound amplicon length, only 12 genera failed to be distinguished by a combination of V36-F348-R1050 and V47-F528-R1164, neither one of which is the best primer pair (V36-F345-R1052).

### Laboratory experiments with designed primers

The best designed primers were tested in the laboratory. Needless to say, the performance of the primers will depend on the microbial composition of the samples on which these primers are applied. We tested these on DNA extracted from lung biolavage samples from the lungs of 8 (unidentified) individuals. The experiments were performed in the Mathee laboratory. PCR was performed on the extracted DNA and gel electrophoresis was used to isolate the amplicons of the appropriate length. The results were compared using BioAnalyzer (http://www.genomics.agilent.com/).

**Figure 1:**
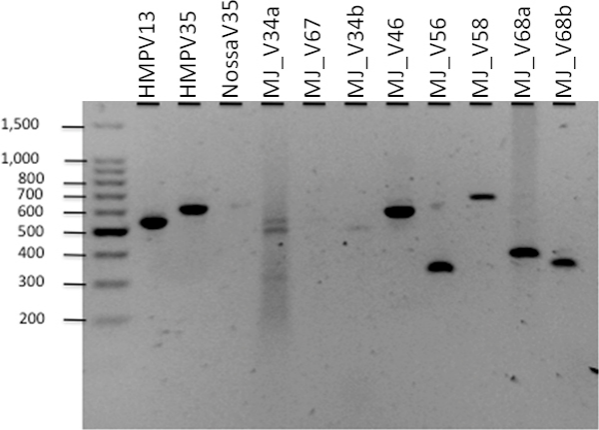
**Comparing HMP and MJ primers**: PCR amplification for one sample with 3 known HMP primer pairs and 8 newly designed primer pairs.

**Figure 2:**
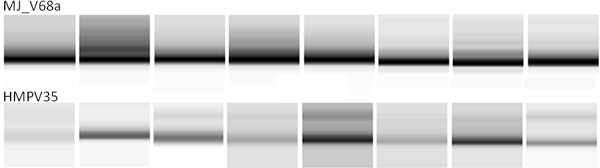
**Comparing HMP and MJ primers**: BioAnalyzer read length distribution for 8 different samples. Top row is for MJV68 and bottom one is for HMPV35.

Figure 1 shows gel images for a sample from a single patient with 11 different primers - 3 HMP primers and 8 new primers from this paper. Under identical cnditions, the figure shows that 5 out of the 8 newly designed primers and 2 of the 3 HMP primers produced sufficient and comparable amplification. We then picked the best performing HMP primer and a MJ primer and tested them with 8 different patient samples. The read length distribution from the BioAnalyzer results in Figure 2 shows that the MJ primer produced more amplification than the HMP primer for most samples. More detailed analyses (not reported here) show that new genera (Alterococcus, Coxiella, Isosphaera, Leptolinea, Rubritalea, and Zavarzinella) and phyla (Armatimonadetes and Lentisphaerae) were detected by the new primers.

## Discussion

The design paramater values shown in Table 3 are default values as suggested in the literature by PCR practitioners. However, the algorithm allows the user to change these values as they see fit. 16S rRNA variable regions are not uniformly variable across all species, i.e., for a group of species a particular variable region might be more conserved than others. Clearly, this differential variability of the regions affects their impact on the choice of primers and the ability of the primers to generate distinguishable amplicons.

Although intuitively it might seem that the greater the span of hypervariable regions of the amplicons from a primer pair, the greater would be its distinguishability, in reality it also depends on the specific taxa matched by the pair (*F*, *R*). A pair (*F*_1_, *R*_1_) with fewer hits than pair (*F*_2_, *R*_2_), and whose amplicon is contained in the amplicon for (*F*_2_, *R*_2_) is likely to distinguish more taxa. Different 16S rRNA regions have different degrees of variability. However, it has been noted that the set of taxa distinguished by two different regions may vary substantially, thus providing a basis for combining primer pairs. The primer pairs that result in the most number of distinguishable taxa is not necessarily the result of combining primer pairs with the best individual results. To summarize, in order to obtain as complete a picture of a microbiome as possible the number of uniquely identifiable extracted species must be maximized. In other words what is needed are compatible forward and reverse primers that bind well to the same, large number of identifiable species, and produce amplicons that are species specific. Taxon distinguishability depends on (1) how many hits a forward/reverse primer has (2) how many hits a primer pair has (3) how variable is the region a primer pair flanks (4) how many primer pairs distinguish different taxa and (5) how many additional taxa can be distinguished by combining two (or more) primer pairs.

In comclusion, we have developed an algorithm that takes as input a set of user-defined values for the primer design parameters and the sequence database, and outputs customized primer pairs that attempt to maximize their ability to produce distinguishable amplicons for the given database. *In silico *experiments prove that the newly designed primers are better than the primers currently used by practitioners. Laboratory experiments confirm the efficacy of the newly designed primers. This tool will positively impact the design of primers for the *Human Microbiome Project *and other related metagenomics projects.

## Competing interests

The authors declare that they have no competing interests.

## Authors' contributions

MJ performed all the computational work and analyses. MJ and GN wrote the paper. JS, ESH, and LS helped with testing the primers in the Mathee laboratory. All authors reviewed the paper.

## Dedication

Sadly we announce the untimely passing of Melita Jaric and dedicate this paper to her memory.

## Declaration

Publication of this work was supported by a grant (KM and GN) from the Florida Department of Health and support from Florida International University.

This article has been published as part of *BMC Proceedings *Volume 7 Supplement 7, 2013: Proceedings of the Great Lakes Bioinformatics Conference 2013. The full contents of the supplement are available online at http://www.biomedcentral.com/bmcproc/supplements/7/S7.
